# Mutation of Cultural Information on the Use of Plant Complexes in Local Medical Systems

**DOI:** 10.1155/2020/7630434

**Published:** 2020-06-23

**Authors:** Janilo I. M. Dantas, André L. B. Nascimento, Taline C. Silva, Ulysses Paulino Albuquerque

**Affiliations:** ^1^Laboratório de Ecologia e Evolução de Sistemas Socioecológicos, Centro de Biociências, Universidade Federal de Pernambuco, Cidade Universitária, Recife-PE 50730-120, Brazil; ^2^Departamento de Biologia, Universidade Federal do Maranhão, Campus III-Bacabal, Avenida João Alberto S/N, Bacabal-MA 65700-000, Brazil; ^3^Grupo de Pesquisa em Etnobiologia e Conservação de Ecossistemas Nordestinos, Departamento de Ciências Biológicas, Universidade Estadual de Alagoas, Palmeira dos Índios-AL 57604-595, Brazil

## Abstract

Despite being an affable strategy of adaptive expectation, the transmission of cultural information can result in unintended changes in the information. This is known as “mutation” in the theory of cultural evolution. The occurrence of information mutations in local medical systems may be greater in some situations. For example, “vegetable complexes” can be used as good study models to show a greater accumulation of mutations due to the variation in the mixtures and combinations of information. Here, we tested the following hypotheses: (H1) medicinal plants in plant complexes generate a greater accumulation of mutations than isolated plants in local medical systems; (H2) information on the medicinal function of the plant species generates a greater proportion of mutations than information on the parts of plants used medicinally; (H3) plants in plant complexes perceived as less efficient undergo more information mutational events; and (H4) changes in information on plant complexes are more random (mutation) than intentional (guided variation). We conducted the study in the Lagoa do Junco community, state of Alagoas, Northeast Brazil. For data collection, we used semistructured interviews to address the use of isolated medicinal plants and plant complexes. Additionally, we assessed the informants' perceptions about the effectiveness of the plants used in these preparations. We found that the mutation rate was higher when isolated plants were used than when plant complexes were used (*p*=0.02), and it was also higher for function than for parts of the medicinal plants (*p* < 0.001). No relationship between the mutations and perceived efficiency of the plants (*p*=0.19) was observed, and changes in information were more random (mutation) than intentional (guided variation) (*p* < 0.001). From an evolutionary perspective, greatly varying information, such as that on plant complexes, did not explain a greater accumulation of mutations. Thus, we suggested that further studies that include other evolutionary parameters that may cause the accumulation of information mutations must be conducted.

## 1. Introduction

The use of plants for medicinal purposes is considered one of the main strategies adopted by human populations to cure or alleviate various diseases [1, 2]. This shows that the existence of diseases has led people to experiment with natural resources present in the environment, contributing to the formation of local medical systems [3]. In this context, the local medical system is a set of perceptions that humans have about diseases and the methods and resources they use to treat health problems [3, 4].

In local medical systems, isolated medicinal plants or plant mixtures or preparations, herein referred to as plant complexes, can be used. The plant complexes include the “bottled,” “syrups,” and “lickers” [5]. Various local populations produce these complexes as home remedies with the function of curing or alleviating one or more diseases [6, 7]. The use of plant complexes dates back many centuries [6]. The practice of using them preferentially for the treatment of diseases, even in the presence of other local options, has become popular [8–10].

The use and production of plant complexes in local medical systems result from the social transmission of information among individuals [8, 11]. However, cultural information is not always reliably transmitted, allowing errors to occur often [12, 13]. Thus, according to the theory of cultural evolution, errors that occur during the transmission of cultural information are called cultural mutations [14]. The errors that occur randomly during the transmission of cultural information are called “information mutations” (information is changed unintentionally) [14]. On the other hand, the errors that occur when information is intentionally altered by individuals are called “guided variations” [14]. Therefore, although the transmission of cultural information plays a key role in cultural evolution, the emergence of certain mutations can lead to failed exchanges of information in social systems because the occurrence of these mutations implies the establishment of behaviors that do not contribute to individual survival (poorly adapted cultural traits) [12, 14, 15].

In the case of information mutations, since local medical systems are characterized as social information systems permeated by the exchange of cultural information [12], the establishment of this process in these systems depends on a set of main factors including (I) incomplete transmission of information [13] (only parts of complex or varied information can be transmitted or assimilated in the minds of individuals); (II) concealment of information [12] (the information transmitted socially among individuals is not always verified after being acquired, favoring people to learn what to do without understanding the reasons for it) [16]; and (III) confusion of the types of information (although the human mind is largely adaptive, its associative authenticity can be counterproductive, leading people to transmit probable information instead of adequate information) [17].

Based on the aspects mentioned above, information mutations may occur when plant complexes are used in local medical systems. These preparations are produced using a combination of widely varying information on medicinal plants and other specific elements [9, 18] in a single event of cultural information transmission, implying a greater probability of the occurrence of information mutations. Thus, in this work, we sought to investigate whether plant complexes functioned as points of accumulation of information mutations in local medical systems. For this, we tested four hypotheses concerning the process of mutation of information. The first hypothesis (*H1*): the use of medicinal plants in plant complexes tends to generate a greater accumulation of information mutations than the use of isolated plants in local medical systems. Additionally, we expected other factors in the local medical system to contribute to the greater occurrence of information mutations. The second hypothesis (H2): information on the medicinal function of plant species generates a greater amount of mutations than information on the parts of plants used as medicine. In local medical systems in the caatinga environment (seasonal dry forest), the information on the medicinal function of plants tends to vary more than the information on the parts of plant species used by individuals. When this occurs, the use of several plants in plant complexes can cause a “causal mismatch” (unintentional combination of information) [19]. When several plants are used together, there may be a lack of understanding of the real medicinal function of the plants that are included in these preparations.

Considering the evidence that, over time, individuals began memorizing more relevant information from an adaptive point of view due to the limited capacity of the human brain to store complex information about the environment [20], we proposed the third hypothesis (H3): medicinal plants in plant complexes perceived as less efficient suffered the highest number of information mutational events. Furthermore, we proposed the fourth hypothesis (H4): information changes that occur when plant complexes are used are more random (mutation) than intentional (guided variation). Learning information from others, compared to intentionally creating, innovating, or changing information (guided variation) [21], is a faster and less costly adaptive strategy [22].

## 2. Materials and Methods

### 2.1. Study Area

The study was conducted in the “Lagoa do Junco” community located in the municipality of Santana do Ipanema in the state of Alagoas, Northeast Brazil [23]. The municipality of Santana do Ipanema has a population of 48,232 inhabitants and is located in the mesoregion of the Sertão Alagoano, 207 km from the state capital Maceió [23]. “Lagoa do Junco” has a semiurban population, characterized as one of the largest urban and popular neighborhoods in the municipality. Totally 188 individuals (63 families), of which 144 individuals were over 18 years old, resided in this community. In the locality, there are some commercial establishments and public spaces, such as a school, a municipal creche, churches, and a municipal health post, in which individuals from the community can access health services. Additionally, a forest, from where individuals obtain and use natural resources and medicinal plants, surrounds the community. This community was selected for the study because it is comprised of people who produce plant complexes that are used traditionally and supplied to local markets and other neighboring locations in the region.

### 2.2. Ethical and Legal Aspects

The realization of this study included instructions from the Resolution (466/12) of the National Health Council for research with human beings. The study proposal was sent to the Research Ethics Committee (CEP) and approved under number CAAE: 97380918.9.0000.5207 of the University of Pernambuco (UPE). Additionally, it was submitted to the Biodiversity Authorization and Information System (SISBIO), which provided proof of registration under number 64841-1 for the collection of botanical material from the study site. Those who agreed to collaborate with the data in this study were invited to sign the Free and Informed Consent Form (ICF).

### 2.3. Data Collection

The data for this study were obtained from September 2018 to June 2019. For data collection, we conducted semistructured interviews [24] of all people who were over 18 years old and agreed to participate in the research. Thus, 120 people were interviewed (corresponding to 82% of the adult population). To investigate their knowledge on local medicinal plants, we applied the free listing technique [24], according to which the informants were asked to list the names of all plants, used for medicinal purposes, known to them. Following that, we conducted semistructured interviews to address the use of plants individually and in plant complexes. For each plant mentioned by the informants, we asked certain questions including the following: (1) For what disease or diseases is this plant indicated? (2) What are the symptoms related to this health problem? (3) What parts of the plant are used for the treatment? (4) How is the medicine prepared? (5) Of the plants that were mentioned, are any of them used in association with others? (6) If so, what other plants are used? (7) What health problems can be treated using this mixture? (8) From whom did you obtain this knowledge?

### 2.4. Plant Collection and Identification

Following the semistructured interviews, the guided tour technique [24] was used to collect the botanical material, and the informants were invited to indicate the medicinal plants that were within or near their properties. For this, we collected specimens with their reproductive materials and identified the collected material with the help of botanical specialists. Exsiccated specimens were deposited at the Institute of Agricultural Research of Pernambuco-IPA.

### 2.5. Local Perception of the Efficiency of Medicinal Plants

After conducting semistructured interviews, we conducted a new stage of data collection in the community. Individual forms were provided to each interviewee, who mentioned using plant complexes, to collect information related to the perceived effectiveness of medicinal plants. We asked each participant to place the plants used in each type of plant complex in the order of their efficiency in treating the indicated diseases, and this resulted in the classification of more and less efficient plants. Additionally, we checked whether possible changes in information were random (mutation) or intentional (guided variation). For this, we assessed the information obtained from individual apprentices (person who acquired information from a specific individual in the local medical system) and transmitters (person who was mentioned as a transmitter of information by the individual apprentices in the local medical system).

### 2.6. Classification of Information Mutation and Guided Variation

To identify the presence of possible information mutations, we compared the information units (IUs) obtained from the person who learned the information with those obtained from the person who transmitted the information. An IU was the association of the therapeutic target with the plant species and the part of the plant used [25]. For example, “pain–*Lippia alba*–leaf,” “pain–*Myracrodruon urundeuva*–bark,” and “cut–*Myracrodruon urundeuva*–leaf.” For this assessment, we analyzed and compared the data collected in the interviews of the apprentices with that of the transmitters. Mutation was considered when: (1)—the therapeutic target (disease) within the IU indicated by the apprentice was different from that indicated by the transmitter and (2)—when the part of the plant used within the IU indicated by the apprentice was different from that indicated by the transmitter. To avoid misinterpretation of the local therapeutic indications, we referred to the symptoms of each disease mentioned by the informants.

Each individual apprentice and transmitter, who presented divergent information, was notified of their information transmitted during the semistructured interviews. We then asked some inductive questions including the following. (1) You mentioned that you use this mixture to cure the disease X. However, can this mixture be used to cure another type of disease? If so, which one? (2) Have you ever used this mixture to cure another disease in the past? If so, which one? (3) You mentioned that you use parts of certain plants X and Y in this preparation. However, can other parts of plants X and Y be used in this preparation? If so, which ones? It was then possible to compare the IUs obtained from the person who learned the information with those obtained from the person who transmitted the information, verifying whether the divergence was random (mutation) or intentional (guided variation).

### 2.7. Data Analysis

To assess whether information mutations occurred more frequently in plant complexes than in isolated plants, we calculated the number of times information mutations occurred in individual plants and plant complexes. Then, the average mutation rates for individual plants and plant complexes and the difference in the averages were calculated. Finally, using the Monte Carlo technique, we created a hypothetical null scenario, in which the differences between the averages were simulated 1000 times at random to obtain greater credibility of the statistical results. The differences between the real averages were considered significant when the probability of occurrence was less than the random probability generated by the null scenario (*p* < 0.05). We used the same procedure to compare (1) the average mutation rate with the average guided variations in plant complexes; (2) the average mutation rate considering the part of the plant used with the average mutation rate considering the medicinal function (treated disease).

To determine whether the perceived efficiency of plants in the plant complex affects the occurrence of mutation, we developed a generalized linear model (GLM) using the binomial family. The dependent variable was the occurrence (1) [or not (0)] of mutation of cultural information, while the independent variable was the value of the order of the perceived efficiency of the plant. Considering that each plant complex has a different size (variety in the number of plants inserted in each complex), a factor that directly affected the calculation of the effect of the independent variable in a multilevel GLM was used to consider the variable differences of the complexes. Thus, we verified the effect of efficiency on the mutation rate of random information of each plant complex and tested the existence of an explanatory trend in the GLM, compared with the null model, using ANOVA analysis. All analyses were performed in the *R* development environment [26] using “lme4” package [27] for multilevel analysis.

## 3. Results

In the present study, we identified 52 medicinal plant species used alone or in association (Table 1). Of the 120 individuals interviewed, 108 mentioned that they produced or used some type of plant complex. We identified and divided 141 types of plant complexes (Table 2) into five categories: 7 types of medicinal baths (plants immersed in water to obtain decoctions to be used topically), 39 types of teas (plants immersed in water to obtain decoctions to be used orally), 26 types of bottled medicine (plants mixed with alcoholic or sweetened substances to form a preparation to be used through closed containers), 64 types of *“lambedor”* (plants immersed in sweetened substances to obtain decoctions), and 5 types of syrups (plants immersed in honey to obtain decoctions) (Table 2).

Contrary to our expectations, the results showed a higher mutation rate in isolated plants than in plant complexes; hence, the average mutation rate was significantly higher (*p*=0.02) for isolated plants (mean = 0.45; standard deviation (SD) = 0.89) than for plant complexes (mean = 0.24; SD = 0.54). Additionally, our analysis showed that the average mutation rate was significantly higher (*p* < 0.001) for function (mean = 0.56; SD = 0.99) than for plant parts (Mean = 0.11; SD = 0.4).

The results indicated that the perceived efficiency of plants in plant complexes was not a reliable variable to explain the existence of information mutations because the inclusion of this variable did not generate a better explanatory model than the null model (*χ*2 = 1.71; *p*=0.19). Thus, perceived efficiency was not a factor that affected the information mutation rates (Table 3). We also observed that the mean of change was significantly higher (*p* < 0.001) for mutations (mean = 0.24; SD = 0.52) than for guided variations (mean = 0.03; SD = 0.16).

## 4. Discussion

### 4.1. Does the Use of Medicinal Plants in Plant Complexes Cause a Greater Accumulation of Information Mutations than the Use of Isolated Plants?

In general, some studies [28, 29] have shown that many of the plant complexes used in traditional medicine consist of various medicinal plants. We expected that this fact would make the transmitted information more susceptible to changes during the information transmission process. However, the information on these preparations was more conservative than the information on isolated medicinal plants.

Studies that specifically address the different types of individual learning are necessary to explain the mechanisms that may cause information on plant complexes to become more conservative than information on isolated medicinal plants. These mechanisms include obtaining information on (I) isolated medicinal plants and (II) plant complexes. For instance, since plant complexes are produced using a combination of plants and specific elements, information on these preparations could be acquired from “prestigious individuals” in the local medical system. Prestigious individuals have a high social status due to their personal experiences and generally become models for other people who imitate their behaviors and follow their traditional customs and knowledge. In this case, information on plant complexes is less susceptible to variation because there would be no other sources “competing” for the transmission of the same information. In contrast, the acquisition of information on isolated medicinal plants can take place through different learning pathways, such as from parents to children (vertical), between individuals of the same generation (horizontal), and between individuals with no kinship (oblique) [14]. This would increase the chances of inheritance of information with mutations [21].

It is important to consider the finding that, over time, due to great information diversity, human memory systems have evolved, allowing certain information that is more relevant from the adaptive point of view to be more easily memorized [30]. Thus, we suggested the need for further studies that will assess the differences in the perceived importance of the use of isolated medicinal plants and plant complexes. If the use of plant complexes is perceived more important, this can be considered a determining factor of the information on these preparations being more conservative.

### 4.2. Does the Information on the Medicinal Function of Plant Species Generate a Greater Amount of Mutations than Information on the Parts of Plants Used Medicinally?

The lower mutation rate for information on the parts of plants used medicinally could be explained by the pattern of use of plant species in the local medical system because only stem, bark, and leaves of plants were used medicinally. Thus, since several plants are used together in plant complexes, there may be a lack of understanding of the real purpose of each plant part in these preparations or that transmitted information was more susceptible to judgment errors [17].

### 4.3. Do Medicinal Plants in Plant Complexes Perceived as Less Efficient by People Suffer the Highest Amount of Information Mutational Events?

Over time, individuals had to filter out information relevant to their survival due to the limited capacity of the human brain to store complex information about the environment [20, 30]. Thus, we expected that the information on plants that were considered more efficient by informants would be more conservative than that on plants that were considered less efficient by informants. However, although some plants in the local medical system were perceived as more efficient than other plants, the perceived efficiency did not influence the occurrence of information mutations. Thus, modification of information occurred, regardless of the importance of the information.

### 4.4. Are Changes in Information on the Use of Plant Complexes More Random (Mutation) than Intentional (Guided Variation)?

Our results indicated that the changes in information occurring in the system were random, and thus, information transmission was an uncontrollable process [14]. This randomness occurred due to the following mechanisms: (I) concealment of information [12]—information transmitted socially among individuals was not verified, since the people enjoyed transmitting information to other individuals, regardless of whether the information was correct or altered [12, 16]; (II) incomplete transmission of information [13]—since plant complexes are produced using a wide variety of plants and specific elements, only parts of the information on these preparations were memorized and transmitted; and (III) confusion of the different types of information—due to the great variety or complexity of information, altered and/or inadequate information was transmitted [17].

It was found that the exchange of cultural information among individuals was often associated with “trade-offs” (cost and benefit) [21]. Although guided variation favored individuals to adapt to their personal experiences better, learning information from others, compared to intentionally creating, innovating, or changing information, is a faster, more adaptive, and less costly strategy [22]. Therefore, there is a need for further studies that specifically address the preferences of the people in the local medical system between obtaining information and creating their own information.

## 5. Conclusions

This study highlighted the occurrence of information mutations in local medical systems and showed that the transmission of knowledge on medicinal plants was one of the main factors of this process. Consequently, it may contribute to the establishment of poorly adapted local cultural traits. However, we found that greatly varying information that must be transmitted did not explain the greater accumulation of information mutations because the occurrence of greater or fewer information mutations might be influenced by other evolutionary factors during the transmission of knowledge. This requires further scientific investigation. Therefore, we suggested that future studies that address the establishment of information mutations in local medical systems and the understanding of this evolutionary process by considering other responsible parameters, such as perceived importance and cultural validation, must be conducted. Accumulation of maladaptive information in local medical systems must be studied further.

### 5.1. Limitations

This study has some limitations. We did not have specific questions for people experienced with medicinal plants and plant complexes, and this made it impossible to identify prestigious individuals. Consequently, a robust analysis of the relationship between the acquisition of information from these individuals and the occurrence of mutations could not be performed. Thus, we suggested that future studies that assess whether the transmission of information from these individuals significantly influences the occurrence of mutations in the system must be conducted.

## Figures and Tables

**Table 1:** Plants used for medicinal purposes were isolated and as plant complexes by individuals from the Lagoa do Junco community, Santana do Ipanema, Alagoas, NE Brazil.

Common name	Latin name	Botanic family	Voucher
Aroeira	*Myracrodruon urundeuva* Allemão	Anacardiaceae	Dantas, JIM929563
Seriguela	*Spondias purpurea* L.	Anacardiaceae	Dantas, JIM 92947
Babosa	*Aloe vera* (L.) Burm.f.	Asphodelaceae	Dantas, JIM Estéril
Grajaú	*Fridericia chica* (Humb. & Bonpl.) L.G.Lohmann	Bignoniaceae	Dantas, JIM Estéril
Umburana	*Commiphora leptophloeos* (Mart.) J.B.Gillett	Burseraceae	Dantas, JIM 92951
Rabo de Raposa	*Harrisia adscendens* (Gurke) Britton & Rose	Cactaceae	Dantas, JIM 93420
Muçambê	*Tarenaya spinosa* (Jacq.) Raf.	Capparaceae	Dantas, JIM 92702
Pratudo	*Kalanchoe cf. crenata* (Andrews) Haw.	Crassulaceae	Dantas, JIM 92699
Bom Nome	*Monteverdia rígida* (Mart.) Biral	Celastraceae	Dantas, JIM 92952
Melão de São Caetano	*Momordica charantia* L.	Cucurbitaceae	Dantas, JIM 92696
Pião Roxo	*Jatropha gossypiifolia* L.	Euphorbiaceae	Dantas, JIM 92700
Quebra Pedra	*Phyllanthus amarus* Schumach.	Euphorbiaceae	Dantas, JIM 92956
Carrapateira (Mamona)	*Ricinus communis* L.	Euphorbiaceae	Dantas, JIM 92705
Hortelã da Folha Pequena	*Mentha* × *villosa* Huds.	Lamiaceae	Dantas, JIM 92949
Sambacaitá	*Mesosphaerum pectinatum* (L.) Kuntze	Lamiaceae	Dantas, JIM 929562
Manjericão	*Ocimum americanum* L.	Lamiaceae	Dantas, JIM 92948
Hortelã da Folha Grande	*Plectranthus amboinicus* (Lour.) Spreng.	Lamiaceae	Dantas, JIM 929560
Boldo	*Plectranthus ornatus* Codd.	Lamiaceae	Dantas, JIM 949561
Alecrim	*Rosmarinus officinalis* L.	Lamiaceae	Dantas, JIM 949510
Mororó	*Bauhinia cheilantha* (Bong.) Steud.	Leg. Caes	Dantas, JIM 92953
Jatobá	*Hymenaea courbaril* L.	Leg. Caes	Dantas, JIM 93419
Catingueira	*Poincianella pyramidalis* (Tul.) L.P.Queiroz	Leg. Caes	Dantas, JIM 92944
Angico	*Anadenanthera colubrina* var. *cebil* (Griseb.) Altschul	Leg. Mim.	Dantas, JIM 92955
Tamarindo	*Tamarindus indica* L.	Leg. Mim.	Dantas, JIM 92701
Mulungú	*Erythrina velutina* Willd.	Leg. Pap.	Dantas, JIM 92959
Romã	*Punica granatum* L.	Lythraceae	Dantas, JIM92697
Acerola	*Malpighia emarginata* DC.	Malpighiaceae	Dantas, JIM 92945
Hibisco	*Hibiscus rosa-sinensis* L.	Malvaceae	Dantas, JIM 92707
Pitanga	*Eugenia pitanga* L.	Myrtaceae	Dantas, JIM 92703
Goiabeira	*Psidium guajava* L.	Myrtaceae	Dantas, JIM 92706
Capim Santo	*Cymbopogon citratus* (DC.) Stapf	Poaceae	Dantas, JIM 929564
Juazeiro	*Ziziphus cotinifolia* Reissek	Rhamnaceae	Dantas, JIM 92698
Noni	*Morinda citrifolia* L.	Rubiaceae	Dantas, JIM 93422
Pé de Limão	*Citrus* sp.	Rutaceae	Dantas, JIM 92708
Laranjeira	*Citrus x aurantium* L.	Rutaceae	Dantas, JIM 92954
Quixabeira	*Sideroxylon obtusifolium* (Roem. & Schult.) T.D.Penn.	Sapotaceae	Dantas, JIM 92946
Pimenta	*Capsicum frutescens* L.	Solanaceae	Dantas, JIM 93421
Erva Cidreira	*Lippia alba* (Mill.) N.E.Br.	Verbenaceae	Dantas, JIM 92704
Testa de Touro	*Kallstroemia tribuloides* (Mart.) Steud.	Zygophyllaceae	Dantas, JIM 92950
Sabugueiro	*Reproductive material not collected*		
Poeijo	*Reproductive material not collected*		
Pau Darco	*Reproductive material not collected*		
Mastruz	*Reproductive material not collected*		
Jaramataia	*Reproductive material not collected*		
Gengilin	*Reproductive material not collected*		
Gengibre	*Reproductive material not collected*		
Eucalipto	*Reproductive material not collected*		
Endro	*Reproductive material Not collected*		
Colônia	*Reproductive material Not collected*		
Ouricuri	*Reproductive material Not collected*		
Amora	*Reproductive material Not collected*		
Canela	*Reproductive material Not collected*		

**Table 2:** Plants complexes used for medicinal purposes by individuals from the Lagoa do Junco community, Santana do Ipanema, Alagoas, NE Brazil.

Plant complexes	Plants used in the complex	Medicinal function of the complex	Number of people who use it
Bath 1	Eucalipto, Hortelã da folha pequena, and Manjericão	Fever	01
Bath 2	Aroeira and Alecrim	Respiratory fatigue	01
Bath 3	Aroeira and Angico	Fever	01
Bath 4	Mamona and Hortelã da Folha Grande	Fever and diarrhea	02
Bath 5	Hortelã da folha grande and Eucalipto	Fever	01
Bath 6	Pau darco and Pratudo	Fever	01
Tea 1	Capim santo and Erva cidreira	Stress, headache, fever, and diarrhea	07
Tea 2	Goiabeira, Pitanga, and Seriguela	Diarrhea	01
Tea 3	Alecrim, Limão and Hortelã da folha Grande	Flu	01
Tea 4	Hortelã da folha grande and Hortelã da folha pequena	Headache, flu, and nausea	10
Tea 5	Pitanga and Goiabeira	Stress	01
Tea 6	Erva Cidreira, Capim Santo, and Camomila	Insomnia	01
Tea 7	Erva cidreira and Hortelã da Folha Grande	Stress, flu, and fever	03
Tea 8	Hortelã da Folha Grande, Hortelã da folha Pequena, and Sabugueiro	Flu	01
Tea 9	Capim Santo, Hortelã da Folha Grande, Hortelã da Folha Pequena, and Eucalipto	Headache and flu	01
Tea 10	Hortelã da Folha Grande, Hortelã da Folha pequena, Quebra Pedra, and Barbatimão	Flu, prostate, and kidney stone	01
Tea 11	None and Erva Cidreira	Fever	02
Tea 12	Angico and Aroeira	Gastritis	02
Tea 13	Hortelã da Folha Grande, Hortelã da Folha Pequena, and Alecrim	Flu	01
Tea 14	Tamarindo and Hortelã da Folha Grande	Flu	01
Tea 15	Hortelã da Folha Grande and Juazeiro	Flu	01
Tea 16	Mastruz, Hortelã da folha Grande, Hortelã da Folha Pequena, Alecrim, Juazeiro, and Romã	Flu, fever, cough, and headache	01
Tea 17	Hortelã da Folha Grande and Arruda	Flu	01
Tea 18	Sabugueiro and Hortelã da Folha Grande	Fever	01
Tea 19	Alecrim and limão	Flu	01
Tea 20	Eucalipto and Aroeira	Fever	01
Tea 21	Hortelã da Folha Grande and Pratudo	Fever	01
Tea 22	Hortelã da Folha Grande and Alecrim	Flu	01
Tea 23	Arruda and Alecrim	Flu	01
Tea 24	Alecrim, Capim Santo and Boldo	Headache and diarrhea	01
Tea 25	Cana de Macaco and Romã	Fever	01
Tea 26	Boldo, Eucalipto and Manjericão	Fever	01
Tea 27	Alecrim and Eucalipto	Headache	01
Tea 28	Boldo and Eucalipto	Fever	01
Tea 29	Pratudo and Boldo	Diarrhea and fever	02
Tea 30	Pratudo and Hortelã da Folha Grande	Flu	01
Tea 31	Pratudo and Umburana	Flu	01
Tea 32	Pau Darco and Romã	Fever	01
Tea 33	Quixabeira and Pau Darco	Migraine	01
Tea 34	Pau Darco, Quixabeira, and Babosa	Headache and fever	01
Tea 35	Aroeira and Pra tudo	Fever and headache	02
Tea 36	Umburana and Alecrim	Flu	01
Tea 37	Capim Santo and Alecrim	Fever	01
Tea 38	Gengibre and Boldo	Headache and diarrhea	01
Tea 39	Pra tudo and Eucalipto	Fever	01
Bottled medicine 1	Hortelã da Folha Grande, Babosa, Alecrim, and Gengibre	Flu and headache	01
Bottled medicine 2	Hortelã da Folha Grande, Hortelã da Folha pequena, Alecrim, and Mastruz	Flu	01
Bottled medicine 3	Hortelã da Folha Pequena, Pratudo, and Babosa	Flu	01
Bottled medicine 4	Hortelã da Folha Pequena, Hortelã da Folha Grande, Arruda, and Gengibre	Flu	01
Bottled medicine 5	Aroeira, Pau Darco, Cajueiro Roxo, and Umburana	Gastritis	01
Bottled medicine 6	Aroeira, pau Darco, and Umburana	Knock	01
Bottled medicine 7	Pra tudo, Hortelã da Folha pequena, and Laranjeira	Flu and fever	01
Bottled medicine 8	Mastruz, Hortelã da Folha Grande, Hortelã da Folha pequena, alecrim, Romã, and Juazeiro	Cough, inflammation, flu, and fever	01
Bottled medicine 9	Mastruz, Capim Santo, Aroeira, and Angico	Gastritis	01
Bottled medicine 10	Umburana, Angico, Hortelã da Folha Grande, Maracujá de Estralo, Arruda, and Babosa	Flu, gastritis, diarrhea, tiredness, and headache	01
Bottled medicine 11	Aroeira, Alecrim, and Laranjeira	Wounds and infection	01
Bottled medicine 12	Hortelã da Folha Grande, Eucalipto, Juazeiro, Pueijo, and Alecrim	Fever, diarrhea, headache, and flu	01
Bottled medicine 13	Romã, Mastruz, Hortelã da Folha Grande, and Quixabeira	Gastritis	01
Bottled medicine 14	Romã, Hortelã da Folha Grande, and Babosa	Diarrhea	01
Bottled medicine 15	Hortelã da Folha Grande, and Aroeira	Flu and fever	01
Bottled medicine 16	Angico and Aroeira	Flu and diarrhea; headache and fever	03
Bottled medicine 17	Babosa and Hortelã da Folha Grande	Dandruff and headache	02
Bottled medicine 18	Babosa and Aroeira	Gastritis and flu	01
Bottled medicine 19	Alecrim and Pra tudo	Flu and fever	02
Bottled medicine 20	Angico, Aroeira, and Umburana	Fever, migraine, and gastritis	02
Bottled medicine 21	Angico and Hortelã da Folha Grande	Fever	01
Bottled medicine 22	Aroeira, Angico, and Alecrim	Flu, fever, and cough	01
Bottled medicine 23	Babosa and Pra tudo	Gastritis	01
Bottled medicine 24	Angico, Aroeira, and Hortelã da Folha Grande	Flu, headache, and dandruff	02
Bottled medicine 25	Aroeira, Angico, and Gengibre	Flu, headache, and fever	03
Bottled medicine 26	Umburana and Aroeira	Fever	01
“Lambedor” 1	Hortelã da Folha Grande, Hortelã da Folha pequena, and Alho	Cough	01
“Lambedor” 2	Hortelã da Folha Grande and Hortelã da Folha pequena	Flu, cough, and fever	06
“Lambedor” 3	Sambacaitá and Erva Cidreira	General inflammation	01
“Lambedor” 4	Hortelã da Folha Grande, Hortelã da Folha pequena, Alho, and Limão	Flu	01
“Lambedor” 5	Hortelã da Folha Grande, Hortelã da Folha Pequena, and Cebolinha	Stress	01
“Lambedor” 6	Aroeira and Cajueiro Roxo	Uterus inflammation and flu	02
“Lambedor” 7	Muçambê, Catingueira, Hortelã da folha Grande, Maracujá de Estralo, Angico, and Aroeira	Asthma and bronchitis	01
“Lambedor” 8	Hortelã da folha Grande, Hortelã da folha pequena, and Babosa	Flu	01
“Lambedor” 9	Hortelã da Folha Grande, and Babosa	Flu	01
“Lambedor” 10	Hortelã da Folha Grande, Alecrim, and Limão	Flu and fever	02
“Lambedor” 11	Hortelã da Folha Grande, Babosa, and Boldo	Headache and flu	01
“Lambedor” 12	Hortelã da folha Grande, Hortelã da folha pequena Alecrim, and Babosa	Headache	01
“Lambedor” 13	Alecrim, Bom Nome, Hortelã da Folha Grande, and Umburana	Flu and headache	02
“Lambedor” 14	Hortelã da Folha Grande, Hortelã da Folha pequena, and Gengibre	Cough	01
“Lambedor” 15	Mastruz, Gengibre and Hortelã da Folha Grande	Flu	01
“Lambedor” 16	Hortelã da Folha Grande, Hortelã da Folha pequena, Alecrim, Mastruz, Pra Tudo, Boldo, and Eucalipto	Flu and headache	01
“Lambedor” 17	Hortelã da Folha Grande, Hortelã da Folha Pequena, Alecrim, and Boldo	Migraine and dizziness	02
“Lambedor” 18	Hortelã da Folha Grande, Hortelã da Folha pequena, and Pra Tudo	Headache and flu	02
“Lambedor” 19	Hortelã da Folha Grande, Gengibre, Alho, and Arruda	Flu and headache	02
“Lambedor” 20	Laranjeira, Eucalipto, and Capim Santo	Fever	02
“Lambedor” 21	Laranjeira, Eucalipto, Capim Santo, Hortelã da Folha Grande, and Hortelã da Folha pequena	Flu	01
“Lambedor” 22	Hortelã da Folha Grande, Hortelã da Folha pequena, Mastruz, and Muçambê	Fever	01
“Lambedor” 23	Limão, Hortelã da Folha Grande, and Capim Santo	Flu and headache	02
“Lambedor” 24	Hortelã da Folha Grande, Pra Tudo, and Romã	Flu and fever	01
“Lambedor” 25	Pra Tudo, Alecrim, Hortelã da Folha Pequena, and Arruda	Flu	01
“Lambedor” 26	Hortelã da folha Pequena, Aroeira, Alho, and Pra Tudo	Headache, flu, and fever	02
“Lambedor” 27	Erva Cidreira, Hortelã da Folha Grande, Alho, and Cebolinha	Flu	01
“Lambedor” 28	Pra Tudo and Hortelã da Folha Grande	Flu and tiredness	01
“Lambedor” 29	Hortelã da folha pequena and Camomila	Headache	01
“Lambedor” 30	Pra Tudo, Hortelã da Folha Grande, and Alho	Headache	01
“Lambedor” 31	Hortelã da folha Grande and Alecrim	Flu and fever	03
“Lambedor” 32	Muçambê, Juazeiro, Alecrim, and Angico	Flu	01
“Lambedor” 33	Juazeiro, Alecrim, and Angico	Flu and fever	01
“Lambedor” 34	Pra tudo, Mastruz, and Hortelã da Folha Grande	Flu	01
“Lambedor” 35	Hortelã da Folha Grande, Hortelã da Folha Pequena, Erva Cidreira, and Umburana	Headache	01
“Lambedor” 36	Hortelã da Folha Grande, Hortelã da Folha Pequena, Alecrim, Cebolinha, and Gengibre	Cough	01
“Lambedor” 37	Pra tudo, Mastruz, Aroeira, Sambacaitá, Limão, and Eucalipto	Cough, bronchitis, breathlessness, and fever	01
“Lambedor” 38	Mastruz and Capim Santo	Flu and cough	01
“Lambedor” 39	Angico and Muçambê	Cough	01
“Lambedor” 40	Hortelã da Folha Grande, Hortelã da Folha pequena, and Alecrim	Cough, allergy, flu, hemorrhoid, headache, and fever	04
“Lambedor” 41	Alecrim, Endro, Testa de Touro, and Babosa	Flu	01
“Lambedor” 42	Alecrim, Endro, and Testa de Touro	Fever	01
“Lambedor” 43	Hortelã da Folha grande, Hortelã da Folha Pequena, Laranjeira, Pra Tudo, and Limão	Cough	01
“Lambedor” 44	Hortelã da Folha Grande, Hortelã da Folha Pequena Pra Tudo, and Catingueira	Fever	01
“Lambedor” 45	Hortelã da Folha Grande, Hortelã da Folha pequena, Alecrim, Manjericão, and Babosa	Flu, fever, cough, and sore throat	01
“Lambedor” 46	Sambacaitá, Hortelã da Folha Grande, Hortelã da Folha Pequena, and Mastruz	Cough	02
“Lambedor” 47	Erva Doce and Erva Cidreira	Stress	01
“Lambedor” 48	Hortelã da Folha Grande, Hortelã da Folha pequena, Alecrim, Poeijo, Mastruz, and Eucalipto	Flu	01
“Lambedor” 49	Hortelã da Folha Pequena, Eucalipto, Alho, Limão, and Alecrim	Diarrhea	01
“Lambedor” 50	Pra tudo, Hortelã da folha Grande, Hortelã da Folha pequena, Eucalipto, Alho, Limão, and Alecrim	Flu and cough	01
“Lambedor” 51	Pra tudo and Hortelã da Folha Pequena	Flu and headache	02
“Lambedor” 52	Hortelã da Folha Grande, Pra tudo, and Romã	Fever	01
“Lambedor” 53	Alecrim, limão, Boldo, and Hortelã da Folha Pequena	Diarrhea and headache	01
“Lambedor” 54	Hortelã da Folha Grande, Babosa, Alecrim, and Mastruz	Flu and cough	02
“Lambedor” 55	Hortelã da Folha Grande, Babosa, and Eucalipto	Fever and headache	02
“Lambedor” 56	Hortelã da Folha Grande, Babosa, Cana de Macaco, and Eucalipto	Fever and headache	01
“Lambedor” 57	Pra Tudo and Alecrim	Fever and flu	02
“Lambedor” 58	Arruda, Alecrim, and Hortelã da Folha Grande	Flu	01
“Lambedor” 59	Arruda and Hortelã da Folha Grande	Flu	01
“Lambedor” 60	Alecrim and Eucalipto	Headache	01
“Lambedor” 61	Arruda, Alecrim, Jatobá, and Pra Tudo	Flu	01
“Lambedor” 62	Angico, Aroeira, and Pra Tudo	Fever, diarrhea, headache, and flu	01
“Lambedor” 63	Gengibre, Hortelã da Folha Grande, and Aroeira	Flu	01
“Lambedor” 64	Hortelã da Folha Grande, Aroeira, Gengibre, Alecrim, and erva Cidreira	Fever, headache, and flu	01
Syrup 1	Hortelã da Folha Grande, Hortelã da Folha Pequena, and Umburana	Flu	01
Syrup 2	Hortelã da Folha Pequena, and Erva Cidreira	Flu	01
Syrup 3	Hortelã da Folha Grande, Hortelã da Folha Pequena, and Limão	Flu and virosis	01
Syrup 4	Hortelã da Folha Grande, Hortelã da Folha Pequena, and Gengibre	Flu and sore throat	01
Syrup 5	Hortelã da Folha Grande, Gengibre, and Alecrim	Flu	01

**Table 3:** Multilevel logistic generalized linear model showing the association between the efficiency of medicinal plants in plant complexes and the existence of information mutations.

	Null model	Model 1
Fixed effect	Coefficient (standard error)	Coefficient (standard error)
Intercept	−8.57 (1.42)^*∗*^	−9.14 (1.52)^*∗*^
Efficiency	—	0.26 (0.20)
Random effect	Variance (standard deviation)	Variance (standard deviation)
Level 2		
Plant complexes	46.63 (6.83)	44.65 (6.68)
Adjust		
AIC	139.8	140.1

^*∗*^ *p* < 0.05.

## Data Availability

The data used to support the findings of this study are available from the corresponding author upon request.
